# Axonal Degeneration of the Vagus Nerve in Parkinson's Disease—A High-Resolution Ultrasound Study

**DOI:** 10.3389/fneur.2018.00951

**Published:** 2018-11-12

**Authors:** Johann Otto Pelz, Elena Belau, Christopher Fricke, Joseph Classen, David Weise

**Affiliations:** Department of Neurology, Leipzig University Hospital, Leipzig, Germany

**Keywords:** Parkinson's disease, vagus nerve, high-resolution ultrasound, atrophy, axonal degeneration, non-motor symptoms

## Abstract

**Background:** Recent histopathological studies revealed degeneration of the dorsal motor nucleus early in the course of Parkinson's disease (PD). Degeneration of the vagus nerve (VN) axons following neurodegeneration of brainstem vagal nuclei should be detectable by high-resolution ultrasound (HRUS) as a thinning of the VNs.

**Methods:** We measured both VNs cross-sectional area (VN-CSA) of 35 patients with PD and 35 age- and sex-matched healthy controls at the level of the thyroid gland using HRUS.

**Results:** On both sides, the VN-CSA was significantly smaller in PD patients than in controls (right: 2.1 ± 0.4 vs. 2.3 ± 0.5 mm2, left 1.5 ± 0.4 vs. 1.8 ± 0.4 mm2; both *p* < 0.05). There was no correlation between the right or left VN-CSA and age, the Hoehn & Yahr stage, disease duration, the motor part of the Unified Parkinson's Disease Rating Scale score, the Montreal Cognitive Assessment score, or the Non-motor Symptoms Questionnaire, and Scale for Parkinson's disease score including its gastrointestinal domain.

**Conclusions:** These findings provide evidencethat atrophy of the VNs in PD patients can be detected *in-vivo* by HRUS.

## Introduction

In Parkinson's disease (PD) autonomic dysfunction may precede the occurrence of the cardinal motor-symptoms by many years (1). Dysfunction of the gastrointestinal tract is often present in the prodromal phase of PD and results in constipation, gastroparesis or nausea (1). Most parts of the gastrointestinal tract receive their parasympathetic input via the vagus nerves (VN) (2). As shown by Braak and co-workers, the central neurodegenerative process of PD often starts with the deposition of phosphorylated α-synuclein in the vagal dorsal motor nucleus (DMN) from where it spreads to the mid-brain and the cortex (3). Since degeneration of neuronal cell bodies is accompanied by degeneration of their axons (4), the VNs of PD patients might be thinner than in healthy controls. However, morphological changes in (peripheral) nerves due to axonal degeneration are subtle and, therefore, hard to detect *in-vivo*.

We hypothesize that the VNs of PD patients are thinner in comparison with healthy subjects and that this VN atrophy can be detected by high-resolution ultrasound (HRUS).

## Methods

The study was approved by the local ethics committee of Leipzig (reference no.: 251-15-13072015). All subjects gave written informed consent in accordance with the Declaration of Helsinki. According to a power analysis based on previously published ultrasound studies measuring the cross-sectional area (CSA) of peripheral nerves in neurodegenerative disorders like amyotrophic lateral sclerosis (5) we estimated that 34 patients with PD had to be examined to detect a difference in CSA of 15% with a power of 0.9. All participants provided informed and written consent prior to their study enrolment.

### Demographic and clinical data

Between July 2016 and July 2017, we recruited 35 patients with a clinical diagnosis of PD according to the British brain bank criteria and 35 healthy subjects without any symptoms indicative of a neurodegenerative disease including movement disorders. All participants underwent a profound neurological examination. The third part of the Unified Parkinson's Disease Rating Scale (UPDRS-III) was used to assess PD motor symptoms. The German version of the Non-motor Symptoms Questionnaire and Scale for Parkinson's disease (NMS) was used to evaluate non-motor symptoms (6). Cognition was assessed by the Montreal Cognitive Assessment (MoCA).

### High-resolution ultrasound of the vagus nerve and image analysis

All 70 participants were examined with HRUS by an experienced nerve sonographer (JP) using the Esaote MyLab Five system with a 15 MHz transducer (probe LA435). Briefly, each VN was visualized in the axial plane at the level of the thyroid gland, and three native B-mode images were recorded at each side. All measurements were done *offline* and in a blinded fashion, i.e., the assessor was unaware of the subject‘s identity and of his/her disease status using the Viewpoint software (5.6.25.281, General Electric Company). To assess the VN-CSA its contour within the hyperechoic epineural rim was outlined. CSAs were determined at a precision of 0.1 mm^2^. The median of the three VN-CSA measurements was used for statistical analyses (7). Adhering to this HRUS protocol we could recently demonstrate good intra-rater- (intraclass correlation coefficient [ICC] 0.85), inter-rater- (ICC 0.76) and across-ultrasound systems agreements (ICC 0.85) (7).

### Statistical analysis

Statistical analyses were performed with SPSS version 24.0 (IBM Corporation; New York, NY, USA). Differences between groups were calculated either by student's *t*-test (normal distribution), by Mann-Whiney-*U*-test (non-normal distribution), or by chi square test (discrete variables). A multiple linear regression analysis was calculated to examine the selective impact of clinical data on right and left VN-CSA in the PD group. A *p* ≤ 0.05 was considered statistically significant.

## Results

Patient and control group were well-balanced in terms of demographic data (Table 1). In comparison to healthy subjects, patients scored higher in the UPDRS-III, the NMS questionnaire including its gastrointestinal domain and had fewer points in the MoCA. Pallaesthesia as a sensible marker of polyneuropathy did not differ between both groups (Table 1).

**Table 1:** Demographic data and clinical characteristics of patients with Parkinson's disease and healthy controls.

	**PD group**	**Control group**	***p*-value**
Sex (female/male)	16/19	20/15	0.339
Age (in years)	67.7 ± 8.8	67.9 ± 6.2	0.805#
Height (in centimeters)	169.7 ± 7.8	167.7 ± 9.1	0.339^*^
Weight (in kilograms)	73.4 ± 13.0	74.9 ± 14.4	0.644^*^
DBS	8/35 (23%)	–	–
Cardiac arrhythmia	2 (6%)	1 (3%)	–
Diabetes mellitus	4 (13%)	1 (3%)	0.164°
Hoehn & Yahr	2.7 ± 1.0	–	–
UPDRS-III (“on”)	22.8 ± 10.2	0.5 ± 0.7	< 0.001#
PD duration (in years)	10.6 ± 7.2	–	–
MoCA (points)	24.9 ± 3.2	26.9 ± 1.7	0.002^*^
Total NMS Quest (30 items)	7.8 ± 4.4	2.7 ± 1.8	< 0.001#
NMS Quest: gastrointestinal tract domain (8 items)	1.7 ± 1.2	0.3 ± 0.4	< 0.001#
Pallaesthesia at the ankle (mean, …/8)	right 5.5 ± 1.9	right 6.0 ± 1.4	0.334#
	left 5.7 ± 1.9	left 5.9 ± 1.5	0.874#
VN-CSA right (mm2)	2.1 ± 0.4	2.3 ± 0.5	0.049#
VN-CSA left (mm2)	1.5 ± 0.4	1.8 ± 0.4	0.021#
VN-symmetry index	1.4 ± 0.3	1.4 ± 0.3	0.315#

* *student's t-test, #Mann-Whitney-U-test, °chi-square test. DBS, deep brain stimulation. UPDRS-III, motor part of the Unified Parkinson's disease rating scale; MoCA, Montreal Cognitive Assessment; NMS, Quest Non-Motor Symptoms Questionnaire; PD, Parkinson's Disease; VN-CSA, Vagus nerve cross-sectional area; VN-symmetry index, ratio of the right and the left VN-CSA*.

**Figure 1:**
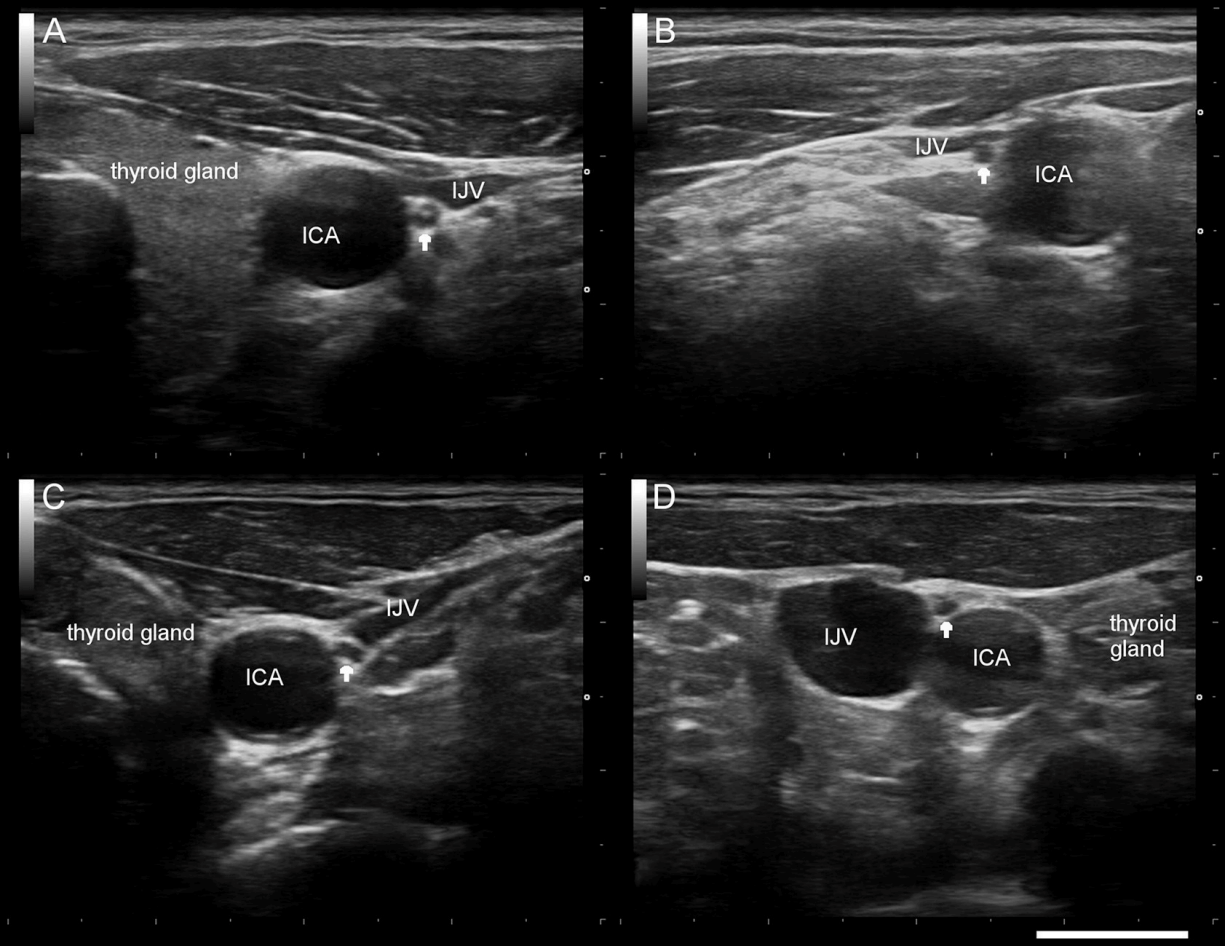
Typical sonographic findings of the right/left vagus nerve (arrow) in a healthy control subject **(A,B)** and a patient with Parkinson's disease **(C,D)**. *ICA* internal carotid artery, *IJV* internal jugular vein. Scale bar 1 cm.

Mean right and left VN-CSA were significantly smaller in PD patients (Figure 1, Table 1). Moreover, in both groups, the left VN-CSA was smaller than the right one (Mann-Whiney-*U*-test, both *p* < 0.01). The VN-symmetry index, that is the ratio between the right and the left VN-CSA, did not differ between groups.

Within the PD group, the multiple regression analysis revealed no association between the right or left VN-CSA and age, Hoehn & Yahr stage, disease duration, UPDRS-III, MoCA score, NMS questionnaire total score or the score of its gastrointestinal domain (Supplementary Table S1).

## Discussion

Although there is growing evidence that the VNs might play a prominent role in the propagation of phosphorylated α-synuclein from the gut to the lower brainstem (1), there are no studies investigating their morphology in PD patients *in vivo*. In the present HRUS study, we demonstrate that both VNs were significantly thinner in PD patients compared to healthy controls. In patients and controls, the right VN-CSA was also significantly larger than the left one confirming our previous findings in 60 healthy subjects (7). This asymmetry may be explained by differential functional topography of left and right brainstem vagal nuclei (8) and the vagal innervation of unpaired abdominal organs. While the right VN predominantly innervates parts of the small intestine, the colon and also contributes to the anterior gastric plexus, the left VN terminates in the anterior gastric plexus, with further branches to the stomach, the liver, and the superior part of the duodenum. Consequential of asymmetric innervation, both VNs also differ in their composition of nerve fibers with the right VN comprising fewer B- and Aδ-nerve fibers (9). However, the VN-symmetry index was similar between both groups suggesting that both VNs were equally affected by axonal degeneration. The VN-CSA reduction by about 9 to 15% is less than previously described in other diseases like amyotrophic lateral sclerosis (15–30% smaller CSA of different peripheral nerves compared to healthy controls) (5). However, the VN consists of axons of multiple brainstem nuclei, namely the nucleus ambiguous, the solitary nucleus, the spinal trigeminal nucleus, and the DMN. Moreover, the VN is composed of around 80% afferent and only 20% efferent fibers (10), although the relative composition of the VN axons stemming from each of its nuclei is not known in detail. Only the DMN mainly contributes to the efferent parasympathetic innervation of enteric neurons (2) and is primarily affected in the early stages of the central neurodegenerative process in PD (3). Hence, the relatively little thinning of the VN may be the consequence of a selective, but almost complete degeneration of DMN axons. Therefore, in those PD cases, where the DMN does not show a prominent phosphorylated α-synuclein pathology, less axonal degeneration should occur and, thus, the VN-CSA might not substantially be reduced. In agreement with these histopathological studies, an electrophysiological study that addressed the integrity of the vagal nuclei complex by recording somatosensory evoked potentials after stimulating the auricular branch of the VN did not point to a prominent dysfunction of the VN's sensory part in PD (11).

We found no correlation between the VN-CSA and disease duration, disease severity or cognitive status. The absent correlations may be explained by an advanced degeneration of the DMN already in early clinical disease stages (that are defined by motor symptoms). There was also no correlation between the VN-CSA and the frequency of non-motor symptoms and in particular with gastrointestinal symptoms as assessed by the NMS questionnaire. However, the qualitative version of the NMS questionnaire might have been not sensitive enough and further studies should employ more specific tests to assess the gastrointestinal function.

One limitation of our study is that we only examined the VNs but no other cranial or peripheral nerves. Thus, we cannot rule out that the CSA reduction is not specific for the VNs. Up to 16% of PD patients have a large fiber peripheral neuropathy (12), which might also involve the VNs. However, neurological examination including assessment of pallaesthesia revealed no difference between PD patients and control group. Therefore, future studies that would validate these findings should include other neurodegenerative disorders, measurements of different peripheral nerves' CSA and neurophysiological as well as laboratory assessment to control for (sub-) clinical peripheral neuropathy and other comorbidities such as vitamin B12, methylmalonic acid or fasting homocysteine levels. Finally, changes in VN-CSA were subtle, thus, 3D ultrasound might help to further improve measurement accuracy (13).

In conclusion, HRUS of the VN as a non-invasive bedside imaging modality revealed thinner VNs in PD patients independent of the disease stage and duration and may therefore be of interest as a biomarker to identify patients at risk of PD (rather than as a longitudinal parameter) in future studies.

## Author contributions

JP and DW designed the experiments. JP and EB performed the experiments. CF gave organizational support. JP and DW analyzed the data. JP, JC, and DW wrote the paper.

### Conflict of interest statement

DW has received speaker honoraria from Ipsen Pharma, Merz Pharmaceuticals, Allergan Inc, UCB Pharma and Abbott. The remaining authors declare that the research was conducted in the absence of any commercial or financial relationships that could be construed as a potential conflict of interest.
