# Supplementary material for: Axonal Degeneration of the Vagus Nerve in Parkinson's Disease—A High-Resolution Ultrasound Study
Source: Front Neurol. 2018 Nov 12;9:951. doi: 10.3389/fneur.2018.00951 (PMC6240697; doi:10.3389/fneur.2018.00951)
Supplement: Supplementary file 1 [file Table_1.DOCX]

**Supplementary table S1.** Correlation between the vagus nerve cross-sectional area

|  | VN-CSA right | VN-CSA left |
| --- | --- | --- |
| Age | *beta* = -0.207  (p = 0.382) | *beta* = 0.096  (p = 0.924) |
| Hoehn & Yahr | *beta* = 0.079  (p = 0.794) | *beta* = -0.045  (p = 0.867) |
| PD duration | *beta* = 0.172  (p = 0.507) | *beta* = 0.395  (p = 0.096) |
| UPDRS-III (“on”) | *beta* = -0.133  (p = 0.619) | *beta* = -0.264  (p = 0.274) |
| MoCA | *beta* = -0.198  (p = 0.345) | *beta* = -0.051  (p = 0.784) |
| Total NMS Quest | *beta* = -0.211  (p = 0.497) | *beta* = 0.015  (p = 0.956) |
| Gastrointestinal tract domain | *beta* = 0.224  (p = 0.464) | *beta* = 0.356  (p = 0.200) |

and clinical data of patients with Parkinson’s disease

A multiple linear regression analysis with the right and left VN-CSA as dependent variables and adjustment for clinical data. *UPDRS-III* motor part of the Unified Parkinson's disease rating scale; *MoCA* Montreal Cognitive Assessment; *NMS Quest* Non-Motor Symptoms Questionnaire*. PD* Parkinson’s Disease; *VN-CSA* Vagus nerve cross-sectional area.
